# Supplementary material for: Circulating and Salivary NGF and BDNF Levels in SARS-CoV-2 Infection: Potential Predictor Biomarkers of COVID-19 Disease—Preliminary Data
Source: J Pers Med. 2022 Nov 9;12(11):1877. doi: 10.3390/jpm12111877 (PMC9697501; doi:10.3390/jpm12111877)
Supplement: Supplementary file 1 [file jpm-12-01877-s001.zip › jpm-1970749-supplementary.pdf]

Journal of
Personalized
Medicine

Supplementary Materials

Table S1. Salivary and serum NGF and BDNF levels.

VariableS	N	ACUTE PHASE					6-MONTHS FOLLOWUP						
		Min	Q1	Median	Q3	Max	Min	Q1	Median	Q3	Max	W	P
Salivary NGF	15	154.8	411.7	797.8	1137.5	3000.6	21.4	70.9	120.0	509.5	1587.6	113	0.00116
Sera NGF	9	4123.4	4341.7	4509.3	4948.1	5358.6	4335.8	4457.9	4640.2	5029.0	7256.0	15	0.42578
Salivary BDNF	15	27.2	77.8	177.0	248.0	580.8	21.7	37.3	44.1	64.9	182.2	114	8.54×10^{-4}
Sera BDNF	9	1791.6	2043.0	2376.6	2757.1	3007.0	1721.8	1808.1	1989.1	2288.0	3637.7	33	0.25

Acute phase: hospitalized patients; Remission phase: 6 months later; Data are expressed as pg/mL; W/P values, non-parametric two-paired Wilcoxon Signed Rank Test; LabTest: duo-set ELISA assay.
